# Evaluation of a clinical risk index for advanced colorectal neoplasia among a North American population of screening age

**DOI:** 10.1186/s12876-015-0395-y

**Published:** 2015-11-19

**Authors:** Arlinda Ruco, David Stock, Robert J. Hilsden, S. Elizabeth McGregor, Lawrence F. Paszat, Refik Saskin, Linda Rabeneck

**Affiliations:** Sunnybrook Health Sciences Centre, Toronto, ON Canada; Department of Medicine, University of Calgary, Calgary, AB Canada; Alberta Health Services – Population, Public & Aboriginal Health, Calgary, AB Canada; Institute for Clinical Evaluative Sciences, Toronto, ON Canada; Prevention and Cancer Control, Cancer Care Ontario, 620 University Avenue, Toronto, M5G 2L7 ON Canada; Department of Medicine, University of Toronto, Toronto, ON Canada; Institute of Health Policy, Management and Evaluation, University of Toronto, Toronto, ON Canada; Dalla Lana School of Public Health, University of Toronto, Toronto, ON Canada

**Keywords:** Colorectal cancer, Colorectal neoplasms, Mass screening, Risk factors

## Abstract

**Background:**

A clinical risk index employing age, sex, family history of colorectal cancer (CRC), smoking history and body mass index (BMI) may be useful for prioritizing screening with colonoscopy. The aim of this study was to conduct an external evaluation of a previously published risk index for advanced neoplasia (AN) in a large, well-characterized cohort.

**Methods:**

Five thousand one hundred thirty-seven asymptomatic persons aged 50 to 74 (54.9 % women) with a mean age (SD) of 58.3 (6.2) years were recruited for the study from a teaching hospital and colorectal cancer screening centre between 2003 and 2011. All participants underwent a complete screening colonoscopy and removal of all polyps. AN was defined as cancer or a tubular adenoma, traditional serrated adenoma (TSA), or sessile serrated adenoma (SSA) with villous characteristics (≥25% villous component), and/or high-grade dysplasia and/or diameter ≥10 mm. Risk scores for each participant were summed to derive an overall score (0–8). The c-statistic was used to measure discriminating ability of the risk index.

**Results:**

The prevalence of AN in the study cohort was 6.8 %. The likelihood of detecting AN increased from 3.6 to 13.1 % for those with a risk score of 1 to 6 respectively. The c-statistic for the multivariable logistic model in our cohort was 0.64 (95 % CI = 0.61–067) indicating modest overlap between risk scores.

**Conclusions:**

The risk index for AN using age, sex, family history, smoking history and BMI was found to be of limited discriminating ability upon external validation. The index requires further refinement to better predict AN in average risk persons of screening age.

## Background

Colorectal cancer (CRC) is one of the most common cancers in women and men worldwide [1, 2]. It is estimated that 136,830 persons were diagnosed with CRC in the United States in 2014 [3]. CRC screening is recommended by the United States Preventive Services Task Force for persons at average risk with annual fecal occult blood test (FOBT), periodic flexible sigmoidoscopy (FS), or colonoscopy [4]. Utilizing easy-to-collect information on clinical risk factors may provide a useful approach in identifying asymptomatic persons who should be referred for colonoscopy rather than a stool test, based on their risk of harboring advanced neoplasia (AN) [5].

Several risk indices or prediction tools for AN or advanced proximal neoplasia (APN) of the colon have been developed [6–20]. These indices have encompassed multiple risk factors for CRC including age, sex, body mass index (BMI), smoking, alcohol, dietary history (red meat consumption), physical activity and in some indices, distal colorectal findings. Intended use for risk indices not employing distal findings include identifying individuals who might be recommended for screening colonoscopy instead of FOBT or fecal immunochemical test (FIT). An example is the work of Kaminski et al. [16], who recently developed a risk index designed to estimate the likelihood of detecting AN at colonoscopy using age, sex, family history of CRC, smoking history and BMI.

We performed an evaluation of the risk index developed by Kaminski et al. [16] by externally validating its performance using a well-characterized cohort of asymptomatic individuals undergoing screening colonoscopy.

## Methods

### The risk score

The risk score for AN developed by Kaminski et al. [16] was derived among a population aged 40 to 66, inclusive, who participated in Poland’s national colonoscopy screening program from January to December 2007, following a recommendation by their family or general practitioner. Candidate predictors of AN included age, sex, BMI, family history of CRC in first-degree relatives, diabetes, smoking history and aspirin use obtained by questionnaire. Based on results of predictive multivariable regression modeling within a test set (*n* = 17,979), the final risk index, as validated in the remainder of the overall screening cohort (*n* = 17,939), comprised age, sex, family history, smoking history in pack-years and BMI. Strength of association from the predictive model determined points assigned for each risk factor. These were summed to derive an overall score ranging from 0 to 8 with the proportion developing AN ranging from 1.3 to 19.1 %, respectively [16].

### Study approval

The study protocol for collection and use of data from our external validation cohort was approved by the research ethics boards at Sunnybrook Health Sciences Centre and Women’s College Hospital in Toronto and the Conjoint Health Research Ethics Board at the University of Calgary.

### Participants

Our external validation cohort was prospectively enrolled from Toronto and Calgary sites using a similar study protocol and data collection methodology as the Veterans Affairs Cooperative Study 380 [21]. From 2003–8, we enrolled asymptomatic persons aged 50 to 74 years referred for outpatient screening colonoscopy by their family doctor to undergo a complete colonoscopy and endoscopic removal of all polyps at Women’s College Hospital in Toronto. In Calgary, using the same inclusion criteria, participants were enrolled from 2009–11 at the Alberta Health Service’s Colon Cancer Screening Centre. Participants were excluded if they: 1) had a prior history of colon surgery, 2) had documented ulcerative colitis, colon polyps, and/or colon cancer, 3) had experienced rectal bleeding in the previous six months on more than one occasion, 4) had a marked change in bowel habits in the previous six months, 5) had lower abdominal pain that would normally require medical attention in the previous six months, 6) had a prior history of sigmoidoscopy, colonoscopy, or barium enema within the past 10 years, or 7) had a medically significant concurrent disease that would preclude the safe performance of colonoscopy as judged by the principal investigator and/or endoscopist.

### Study protocol

Eligible persons, who provided consent, completed a baseline questionnaire that covered demographic information, history of prior colon examinations (sigmoidoscopy, colonoscopy, and barium enema), medical history, prior surgeries, smoking history, alcohol consumption, physical activity, non-steroidal anti-inflammatory drug (NSAID) use, and family history of cancer.

In our external validation cohort, we first assessed the strength of association between the predictors of AN identified by Kaminski et al. [16] in their test cohort. In the main analysis, we used the risk score developed by Kaminski et al. [16] with updated definitions (Table 1) to include participants as old as 74 years of age in our sample. Those older than 66 years were treated the same as those in the highest risk category for age (60–66) and were given a risk score of 3. Risk scores for each participant were summed to derive an overall score. Since our sample did not include any participants younger than 50 years of age, overall scores ranged from 1 to 8 in our sample. Colonoscopy findings were categorized based on the most advanced finding. AN was defined identically to that of Kaminski et al. [16] and included cancer or a tubular adenoma, traditional serrated adenoma (TSA), or sessile serrated adenoma (SSA) with villous characteristics (≥25% villous component), and/or high-grade dysplasia and/or diameter ≥10 mm.

**Table 1:** Risk index adapted from Kaminski et al. [6] with updated definitions to include participants older than 66 years

Risk factor	Category	Score
Age, years	40–49	0
	50–54	1
	55–59	2
	60–66	3
	>66	3
Sex	Female	0
	Male	2
Family history	None	0
	1 first-degree relative ≥ 60 years old	1
	1 first-degree relative < 60 years old	2
	2 first-degree relatives	2
Smoking, pack years	None	0
	<10	0
	10–19	1
	≥20	1
BMI, kg/m^2^	<25	0
	25–29	0
	≥30	1 – Female
		0 – Male

We also conducted a sensitivity analysis to explore the performance of the risk index in a cohort with the same upper age limit as that of Kaminski et al. [16] by repeating all analyses after excluding those older than 66 years.

### Statistical analysis

Descriptive and standard univariate analyses were used to describe baseline characteristics among participants. Logistic regression was used to estimate the strength of association between clinical risk factors identified by Kaminski et al. [16] in their test cohort and AN. A *p*-value of <0.05 was considered statistically significant. The concordance or c-statistic was used to measure ability of the risk index to discriminate between persons with and without AN. The c-statistic can range from 0.5 (discriminating ability equivalent to random chance) to 1.0 (perfect discriminating ability). A c-statistic between 0.7 and 0.8 indicates modest discrimination but not necessarily clinical utility. Criteria considered for clinical utility include the ease with which the information can be collected in a clinical setting and is therefore readily available. All analyses were completed using SAS version 9.3 (SAS Institute, Cary, NC).

## Results

A total of 5137 participants comprised our external validation cohort. Table 2 outlines the distribution of clinical risk factors and colonoscopy findings among the study participants. The mean age (SD) of the cohort was 58.3 (6.2) years and 54.9 % were women (*n* = 2821). More than half (55.5 %) of the participants reported not ever having smoked and approximately a quarter (26.2 %) had an adenoma or cancer (*n* = 1344) when categorized by most advanced finding at colonoscopy. The prevalence of AN in the study cohort was 6.8 %.

**Table 2:** Clinical characteristics and colonoscopy findings of the study cohort (*N* = 5,137)

	All (*N* = 5,137)	Women (*n* = 2,821)	Men (*n* = 2,316)
Age group in years, n (%)			
50–54	1,764 (34.3)	1,016 (36.0)	748 (32.3)
55–59	1,404 (27.3)	731 (25.9)	673 (29.1)
60–66	1,330 (25.9)	734 (26.0)	596 (25.7)
>66	639 (12.4)	340 (12.0)	299 (12.9)
Family History of CRC, n (%)			
None	4,648 (90.5)	2,526 (89.5)	2,122 (91.6)
1 first-degree relative ≥ 60 years old	381 (7.4)	222 (7.9)	159 (6.9)
1 first-degree relative < 60 years old	83 (1.6)	55 (2.0)	28 (1.2)
2 first-degree relatives	25 (0.5)	18 (0.6)	7 (0.3)
Smoking history in pack-years, n (%)			
None	2,849 (55.5)	1,617 (57.3)	1,232 (53.2)
<10	1,342 (26.1)	779 (27.6)	563 (24.3)
10–19	419 (8.2)	204 (7.2)	215 (9.3)
≥20	527 (10.3)	221 (7.8)	306 (13.2)
BMI in kg/m^2^, n (%)			
<25	1,772 (34.5)	1,226 (43.5)	546 (23.6)
25-29	2,149 (41.8)	984 (34.9)	1,165 (50.3)
≥30	1,216 (23.7)	611 (21.7)	605 (26.1)
Most advanced finding at colonoscopy, *n* (%)			
None	3,232 (62.9)	1,943 (68.9)	1,289 (55.7)
Hyperplastic polyp	561 (10.9)	305 (10.8)	256 (11.0)
Non-advanced neoplasia	993 (19.3)	427 (15.1)	566 (24.4)
Advanced neoplasia	351 (6.8)	146 (5.2)	205 (8.9)

*BMI* body mass index

**Fig. 1:**
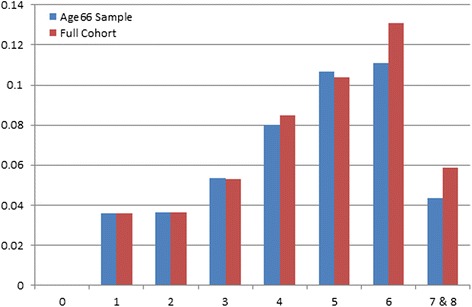
The proportion of advanced neoplasia (AN) by overall risk score in the whole cohort and in those up to 66 years of age

Figure 1 outlines the proportion of AN across overall risk scores. The prevalence of AN increased as the risk scores increased from 1 to 6. Our sample had very few (*n* = 2) participants with a score of 7 or 8 who had AN. The likelihood of detecting AN increased from 3.6 to 13.1 % for those with a risk score of 1 to 6 respectively.

The multivariable-adjusted model (Table 3) shows the adjusted odds ratios (ORs) along with 95 % confidence intervals (CI) and *p*-values for associations between risk index factors and AN. Older age (≥55 years), smoking ≥20 pack-years and a BMI ≥30 were significantly associated with AN. The c-statistic for the multivariable logistic model in our cohort was 0.64 (95 % CI = 0.61–067) indicating modest overlap in probability of predicting AN across risk scores. When those older than 66 years of age were excluded from the cohort (*n* = 644), the performance of the risk index did not materially change (c-statistic 0.64; 95 % CI = 0.60–0.67). The prevalence of AN in the age-restricted sample ranged from 3.6 to 11.1 % for those with risk scores of 1 and 6 respectively (Fig. 1).

**Table 3:** Multivariable-adjusted associations between clinical risk factors and advanced neoplasia

	LR test	Adjusted OR (95 % CI)		Adjusted β coefficient
	*P*-value		*P*-value	
Age group in years	<.001			
50–54		1.0		0
55–59		1.39 (1.03–1.88)	.032	.33
60–66		1.59 (1.18–2.15)	.002	.47
>66		1.95 (1.38–2.76)	<.001	.67
Family History of CRC	.45			
None		1.0		0
1 first-degree relative ≥ 60 years old		1.34 (0.92–1.95)	.13	.29
1 first-degree relative < 60 years old		0.74 (0.27–2.05)	.56	-.30
2 first-degree relatives		1.29 (0.30–5.58)	.73	.25
Sex	<.001			
Female		1.0		0
Male		1.38^a^ (0.89–2.14)	.15	.32
Smoking history in pack-years	.011			
None		1.0		0
<10		1.01 (0.77–1.33)	.94	.098
10–19		1.35 (0.92–1.92)	.12	.30
≥20		1.66 (1.21–2.28)	.002	.51
BMI in kg/m^2^	.006			
<25		1.0		0
25–29		1.02^b^ (0.68–1.53)	.91	.022
≥30		1.60^c^ (1.06–2.40)	.024	.47
BMI-sex interaction	.61			
Male, 25–29 kg/m2		1.88^d^ (1.33–2.64)	.33^f^	.28^f^
Male, ≥30 kg/m2		2.56^e^ (1.76–3.71)	.62^f^	.15^f^

*BMI* body mass index, *CRC* colorectal cancer, *CI* confidence interval, *LR* likelihood ratio, *OR* odds ratio

^a,b,c,d,e^Odds ratio for overall sex-BMI effect: ^a^male, <25 kg/m^2^; ^b^female, 25–29 kg/m^2^; ^c^female, ≥30 kg/m^2^; ^d^male, 25–30 kg/m^2^; ^e^male ≥30 kg/m^2^

^f^β coefficient/*P*-value for sex-BMI multivariable interaction terms

Table 4 outlines the performance characteristics of the risk score in our external validation cohort and includes sensitivity, specificity, positive predictive value (PPV) and negative predictive value (NPV). The PPV (probability of AN given a certain score) for individuals with a risk score of 6, and 7 and 8 (combined) was 12.4 and 5.9 % respectively in our external validation cohort.

**Table 4:** Performance characteristics of the risk score in our cohort (*N* = 5,137)

Risk Score	Persons	AN (n)	Percent	Sensitivity (%)	Specificity (%)	PPV	NPV
1	637	23	3.61	100.00	0.00	6.83	--
2	738	27	3.66	93.45	12.83	7.29	96.39
3	1493	79	5.29	85.75	27.68	8.00	96.36
4	1039	88	8.47	63.25	57.23	9.78	95.50
5	898	93	10.36	38.18	77.10	10.89	94.45
6	298	39	13.09	11.68	93.92	12.35	93.55
7 & 8^a^	34	2	5.88	0.57	99.33	5.88	93.16
Total	5,137	351	6.83				

^a^Due to small sample risk scores 7 and 8 are presented in one row

## Discussion

We report here that the risk index developed by Kaminski et al. [16], when externally validated in a large cohort of 5137 asymptomatic individuals aged 50 to 74 years, was found to be less predictive of AN compared to the Kaminski et al. [16] cohort. Excluding those older than 66 years of age did not materially change this finding. We found that the overall prevalence of AN in our cohort ranged from 3.6 to 13.1 % compared to 4.3 and 13.7 % in the cohort of Kaminski et al. [16] for those with risk scores of 1 and 6 respectively. The prevalence of AN further increased for those with risk scores of 7 or 8 in the Kaminski et al. [16] cohort (19.1 %), while decreasing substantially to 5.9 % in our cohort. The c-statistic in our sample was similar to that of Kaminski et al. [16] for the validation set (0.64 and 0.62 respectively). Associations between smoking, BMI and AN as previously reported in Kaminski et al [16] were also confirmed in our cohort. However, unlike in the test sample of Kaminski et al. [16] we did not observe an association between family history and AN, nor did we find evidence of an interaction between BMI and sex. A possible barrier to applying the risk index developed by Kaminski et al. [16] in clinical practice is the ability to differentiate between the bulk of the patients who fall in the middle of the risk score continuum as a threshold for classifying individuals into low and high risk categories was not specified [22].

Previous studies have found that the performance of risk indices has been limited when validated in separate independent cohorts. For example, Imperiale et al. [6] developed a risk index for APN using information on age, sex and distal colorectal findings. Approximately 67 % of persons with APN were classified as high risk in the derivation subgroup and the c-statistic was 0.81, indicating good to excellent discrimination [6]. Levitzky et al. [23] externally evaluated the risk index of Imperiale at al [6] in a sample of 1481 white, 1329 black and 689 Hispanic participants. The authors reported that the likelihood of having APN was only moderately discriminated by the risk index (c-statistic of 0.63 for blacks, 0.68 for Hispanics, and 0.62 for whites) [23]. We have also recently reported on an evaluation of the risk index of Imperiale et al. [6] using the same cohort described in this study [24]. Similarly to Levitzky et al. [23], we found that the Imperiale et al. [6] risk index did not perform as well in our cohort (c-statistic 0.62; 95 % CI = 0.58–0.66) [24]. If all persons classified in the intermediate and high risk categories were recommended for colonoscopy, only 73.7 % of those with APN would have been identified [24] compared to approximately 92 % in the Imperiale et al. cohort [6].

A review of risk prediction models for colorectal cancer found that no model sufficiently covers all known risk factors for CRC [25]. The authors suggested that a new comprehensive model is needed that is suitable for assessment of people across the full range of risk. However, concerns remain about the addition of risk factors, which may in turn compromise the use of the index in routine clinical practice because of the additional time required to collect the information. Further refinement of these indices may provide more consistent discriminating abilities and the capability to help physicians and their patients make informed decisions regarding screening. Refinement of these indices could include the use of additional risk predictors found to be useful in epidemiological studies, such as waist circumference (WC) instead of or in conjunction with BMI [26]. Recently, a study from the Netherlands reports on a risk prediction model for AN by utilizing quantitative results from the FIT in conjunction with risk factors for AN [27]. This strategy improved discrimination ability significantly compared to a model utilizing FIT results only [27]. Although this strategy must be further validated in a similar study like our own in other independent cohorts, it has the possibility to improve the effectiveness of FIT-based CRC screening, rendering it a prospectively useful tool in the prioritization of colonoscopy resources.

Our findings must be considered in light of the study strengths and limitations. We included information on those older than 66 years. Thus, our cohort of 50 to 74 year olds is representative of an average risk screening population. Excluding those older than 66 years of age did not make a difference to our results and thus strengthens our conclusion. We had a larger proportion of men in our cohort with a more equal distribution of men and women compared to Kaminski et al. [16]. On the other hand, participants in our cohort were recruited from two different sites from outpatient colonoscopy clinics. There is the possibility of selection bias as participants who provided consent may be different from those who decided not to provide consent. The questionnaire used to collect information on risk factors was originally designed in the Veterans Affairs Cooperative Study 380 and then adapted for the Canadian population [21]. As such, there is the possibility that the questions or items were not identical between the two cohorts. In addition, we only had 34 persons with a risk score of 7 or 8, only two of whom had AN, potentially limiting our ability to evaluate the performance of this index for higher risk individuals.

## Conclusions

In conclusion, the risk index for AN using age, sex, family history of CRC, smoking history and BMI, as derived by Kaminski et al. [16] was found to be less predictive of AN in our North American population of screening age. The index requires further evaluation and refinement to better predict AN in average risk persons. However, the index may be useful in very low-resource settings where access to screening colonoscopy is very limited.

## Abbreviations

AN: advanced neoplasia
APN: advanced proximal neoplasia
BMI: body mass index
CI: confidence interval
CRC: colorectal cancer
FIT: fecal immunocheminal test
FOBT: fecal occult blood test
FS: flexible sigmoidoscopy
NPV: negative predictive value
NSAID: non-steroidal anti-inflammatory drug
OR: odds ratio
PPV: positive predictive value
SD: standard deviation
SSA: sessile serrated adenoma
TSA: traditional serrated adenoma
WC: waist circumference
